# Plasma cytokines, chemokines and cellular immune responses in pre-school Nigerian children infected with *Plasmodium falciparum*

**DOI:** 10.1186/1475-2875-12-5

**Published:** 2013-01-07

**Authors:** Cariosa Noone, Michael Parkinson, David J Dowling, Allison Aldridge, Patrick Kirwan, Síle F Molloy, Samuel O Asaolu, Celia Holland, Sandra M O’Neill

**Affiliations:** 1 Parasite Immune Modulation Group, School of Nursing and Human Sciences, Faculty of Science and Health, Dublin City University, Glasnevin Dublin 9, Ireland; 2 Department of Zoology, School of Natural Sciences, Trinity College Dublin, Dublin 2, Ireland; 3 Obafemi Awolowo University, Ile-Ife, Nigeria

**Keywords:** Cytokines, Chemokines, Cellular responses, Plasmodium falciparum, Children, Nigeria, Ascaris lumbricoides

## Abstract

**Background:**

Malaria is a major cause of morbidity and mortality worldwide with over one million deaths annually, particularly in children under five years. This study was the first to examine plasma cytokines, chemokines and cellular immune responses in pre-school Nigerian children infected with *Plasmodium falciparum* from four semi-urban villages near Ile-Ife, Osun State, Nigeria.

**Methods:**

Blood was obtained from 231 children (aged 39–73 months) who were classified according to mean *P. falciparum* density per μl of blood (uninfected (n = 89), low density (<1,000, n = 51), medium density (1,000-10,000, n = 65) and high density (>10,000, n = 22)). IL-12p70, IL-10, Nitric oxide, IFN-γ, TNF, IL-17, IL-4 and TGF-β, C-C chemokine RANTES, MMP-8 and TIMP-1 were measured in plasma. Peripheral blood mononuclear cells were obtained and examined markers of innate immune cells (CD14, CD36, CD56, CD54, CD11c AND HLA-DR). T-cell sub-populations (CD4, CD3 and γδTCR) were intracellularly stained for IL-10, IFN-γ and TNF following polyclonal stimulation or stimulated with malaria parasites. *Ascaris lumbricoides* was endemic in these villages and all data were analysed taking into account the potential impact of bystander helminth infection. All data were analysed using SPSS 15 for windows and in all tests, *p* <0.05 was deemed significant.

**Results:**

The level of *P. falciparum* parasitaemia was positively associated with plasma IL-10 and negatively associated with IL-12p70. The percentage of monocytes was significantly decreased in malaria-infected individuals while malaria parasitaemia was positively associated with increasing percentages of CD54^+^, CD11c^+^ and CD56^+^ cell populations. No association was observed in cytokine expression in mitogen-activated T-cell populations between groups and no malaria specific immune responses were detected. Although *A. lumbricoides* is endemic in these villages, an analysis of the data showed no impact of this helminth infection on *P. falciparum* parasitaemia or on immune responses associated with *P. falciparum* infection.

**Conclusions:**

These findings indicate that Nigerian children infected with *P. falciparum* exhibit immune responses associated with active malaria infection and these responses were positively associated with increased *P. falciparum* parasitaemia.

## Background

*Plasmodium falciparum* malaria accounts for approximately 250–300 billion clinical cases of malaria worldwide and is highly endemic in Africa [1]. Approximately one in every five child deaths in Africa are due to malaria with the risk of cerebral malaria being highest in children aged two to four years. Natural acquired immunity rarely occurs before two years and its development is associated with increasing age, which correlates with a reduction in mortality rates due to the more severe forms of *P. falciparum* infection [2]. It is, therefore, important that immune responses in young children are examined in order to further define immunological association with *P. falciparum* infection.

Malaria infection is predominantly characterized by a T helper 1 (Th1) response and the production of pro-inflammatory cytokines such as IL-12-p70, interferon gamma (IFN-γ) and tumour necrosis factor (TNF). These inflammatory cytokines are considered critical in controlling parasitaemia, especially during the early stages of *P. falciparum* infection [3,4]. Conversely during chronic malaria infection, if these robust inflammatory responses are not tightly regulated, they can lead to immunopathology and severe forms of malaria [5,6]. Regulatory cytokines, including interleukin (IL)-10 and transforming growth factor beta (TGF-β) were shown to be important in dampening down T helper (Th) 1 inflammatory responses associated with immune pathology in the more severe forms of *P. falciparum* infection [5,6]. There also a range of other mediators, such as IL-17, IL-4, nitric oxide, C-C chemokine RANTES, matrix metalloproteinases 8 (MMP8s) and tissue inhibitor of metalloproteinases 1 (TIMP1) that have been linked to disease severity in malaria-infected individuals [7-9].

Malaria infection is strongly influenced by the release of inflammatory mediators from innate immune cells where early interactions between blood-stage parasites and these are critical in controlling parasitaemia and the subsequent elimination of infection [5,6]. Innate immune cells including antigen presenting cells, such as dendritic cells, and macrophages are an early source of pro-inflammatory cytokines, such as IL-12 and TNF. Other innate immune cells such as natural killer cells and γδ-T-cells are an early source of IFN-γ. These cells, through the release of inflammatory mediators and from cell-to-cell contact with naïve T-cells, also shape the adaptive immune response.

This is the first study to assess immune responses during uncomplicated malaria infection in Nigerian pre-school children in four semi-urban villages near Ile-Ife, Osun State, Nigeria [10]. *Plasmodium falciparum* infection is endemic in this region with a high prevalence in pre-school children [11]. Recent studies have also shown a 25% prevalence of *Ascaris lumbricoides* in this cohort [12]. Since helminth infection can impact upon the outcome of malaria infection [13-15] the potential impact of *A. lumbricoides* upon *P. faliciparum* parasitaemia and its associated immune responses were examined.

## Methods

### Study design and participants

231 blood samples were obtained from children at the final time point in a double-blind placebo-controlled randomized trial on children aged 39–73 months in four semi-urban villages, Akinlalu, Ipetumodu, Moro and Edunabon, near Ile-Ife, Osun State, Nigeria. Details of the study area, design and participants were published previously [10]. This current sub-study was to examine immune responses associated with *P. falciparum* infection and examine the impact of *A. lumbricoides*. Data were available on children’s age and infection status for *P. falciparum* and *A. lumbricoides* infection. Children who suffered from severe malaria were treated and excluded from the study and therefore only individuals with uncomplicated malaria were included (malaria parasitaemia and fever >37.5°C) [10]. The study protocol was approved by the Ethics and Research Committee, Obafemi Awolowo University Teaching Hospitals’ Complex, Ile-Ife, Nigeria.

### Isolation of peripheral blood mononuclear cells

Ten ml of blood (in tubes containing heparin) was obtained from 231 children, ranging in age from 39–73 months. Peripheral blood mononuclear cells (PBMCs) and plasma were obtained following histopaque (Sigma-Aldrich, St Louis, MO, USA) density gradient centrifugation. PBMCs were collected and stored in liquid nitrogen and plasma samples were stored at −80°C.

### ELISA

Human IFN-γ, IL-10, TGF-β, TNF, IL-4 and IL-12p70 Opti-EIA kits (BD Biosciences) and human IL-17, RANTES, MMP-8 and TIMP-1 DuoSet ELISA Developmental Kits (R&D, Minneapolis, MN, USA) were used to quantify cytokine, chemokine and metalloproteinase levels in plasma samples as described per manufacturers instructions. NO levels were also measured in plasma using the Greiss Reagent System (Promega, Madison, WI, USA).

### Flow cytometry and *in vitro* culture

The following mAbs were used for cells surface staining and intracellular cytokine staining: FITC-conjugated anti- CD4, CD14, CD36, CD56, IFN-γ; PE-conjugated anti- γδTCR, CD54, IL-10; and APC-conjugated anti- CD3, CD11c, HLA-DR, IL-2 and TNF (eBiosciences, San Diego, CA, USA). Isotype controls included FITC-conjugated mouse IgG1, IgM, IgG2a, IgG2b; PE-conjugated mouse IgG1, IgG2b and APC-conjugated mouse IgG1, IgG2b and rat IgG2a (eBiosciences). PBMCs were thawed and cultured in complete RPMI containing 10% FCS (foetal calf serum), 1% L-glutamine and 1% penicillin/streptomycin solution (Bio-sciences Ltd, Co. Dublin, Ireland). For intracellular cytokine staining, PBMCs were stimulated with 50 ng/ml PMA (Phorbol 12-myristate 13-acetate) and 1 mg/ml ionomycin for 4 h and to block cytokine secretion, 10 mg/ml Brefeldin A (Sigma) was added to the culture media. Cells were then washed and stained with cell surface mAbs, fixed with 4% PFA and permeabilized with 0.2% saponin (Sigma), before incubation with antibodies for IL-12, IFN-γ, IL-10 and TNF cytokines. Appropriately labelled isotype-matched antibodies were used as controls. Acquisition was performed using a FACSCalibur flow cytometer (BD Biosciences), and analysis of results performed using FlowJo software (Tree Star). A sample of gating strategy for T Cells in shown in Additional file 1.

PBMCs (1 × 10^6^ cells/ml) from a cohort of children were also cultured on a 24-well plate with mycoplasma free *P. falciparum* parasites (at a ratio of 1:5), which were extracted from cell culture by saponin lysis (0.15%), were kindly provided by Dr Alison Creasey, University of Edinburgh, Scotland. After three days, cell culture supernatants were harvested and frozen for subsequent measurement of IFN-γ, IL-10, and IL-5 by commercial ELISA.

### Statistical analysis

All data were analysed using SPSS 15 for windows. Percentage data were normalized prior to analysis by Arcsin transformation. Skewed data were normalized prior to analysis by log transformation. For differences between multiple groups, one-way ANOVA with Post-hoc testing by Tukey’s HSD test was used. For data with more than one factor, factorial ANOVA was used. Association between variables was assessed using regression analysis. For differences between two treatments 2-tailed Student *t*-test was used. In all tests, *p* <0.05 was deemed significant.

## Results

### *Plasmodium falciparum* parasitaemia was positively associated with IL-10 and negatively associated with IL-12p70 levels in the plasma of infected children

**Figure 1:**
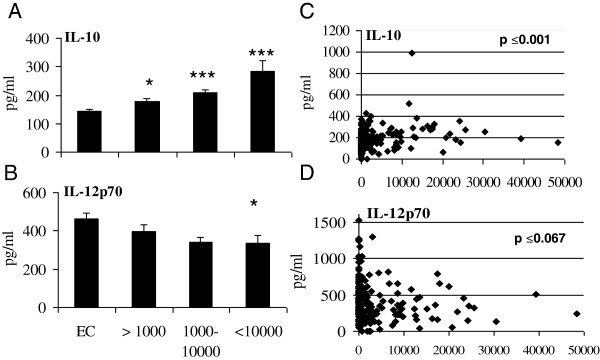
***Plasmodium falciparum *parasitaemia was positively associated with IL-10 and negatively associated with IL-12p70 levels in the plasma of infected children. **Mean plasma levels of **(A)** IL-10 and **(B)** IL-12p70 were determined by ELISA for each group (low (<1,000; n=51), medium (1,000-10,000; n=65) and high (>10,000; n=22) mean parasite density (per μl of blood) and compared to endemic controls (EC) (n=89). ^*^, *p* ≤0.05; ^**^, *p* ≤0.01 and ***, *p* ≤0.001 (ANOVA). Parasitaemia was plotted against **(C)** IL-10 and **(D)** IL-12p70 and association between variables was assessed using regression analysis. *p* <0.05 was deemed significant.

Children infected with *P. falciparum* were divided into groups based upon the mean *P. falciparum* density per μl of blood (uninfected (n = 89), low density (<1,000, n = 51), medium density (1,000-10,000, n = 65) and high density (>10,000, n = 22)) to determine if an association exists between parasitaemia and plasma immune factors (Figure 1A-B, Additional file 2). A reciprocal relationship between IL-10 (low density (p ≤0.01), medium (p ≤0.001) and high density (p ≤0.001) (Figure 1A) and IL-12p70 levels (Figure 1B; high density p ≤0.05) was observed. Regression analysis revealed that increases in IL-10 were significantly associated with increased *P. falciparum* parasitaemia (*P* ≤0.001, Figure 1C), however, IL-12p70 was not negatively associated with *P. falciparum* parasitaemia (*P* ≤0.067, Figure 1D). Nitric oxide, IFN-γ, TNF IL-17, IL-4 and TGF-β, RANTES, MMP-8 and TIMP-1 in plasma was not associated with *P. falciparum* parasitaemia (Additional file 2).

### Malaria infection was associated with a decrease in the percentage of monocytes and enhanced percentages of CD11c^+^, CD54^+^, CD56^+^ but not HLA-DR^+^ and CD36^+^ cell populations

**Figure 2:**
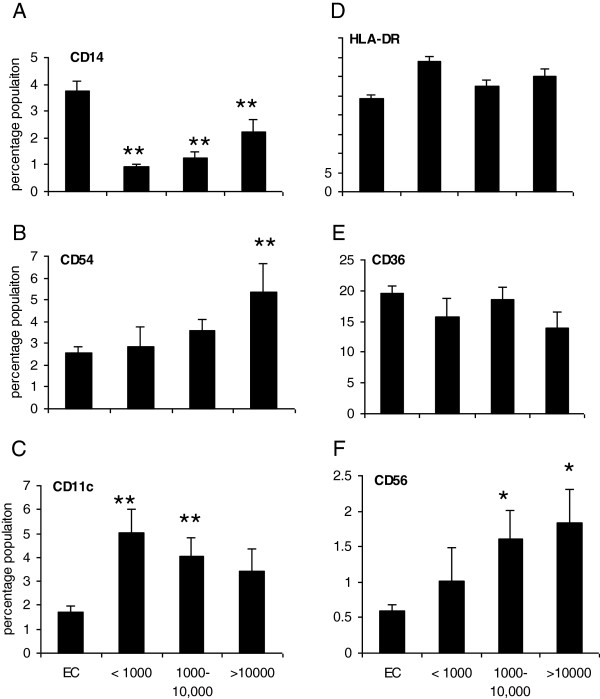
**Malaria infection was associated with decreased CD14**^**+ **^**percentages and enhanced percentages of CD11c**^**+**^**, CD54**^**+**^**, CD56 but not HLA-DR**^**+ **^**and CD36**^**+ **^**cell populations. **PBMCs were stained extracellularly for CD14 **(A)**, CD54 **(B)** CD11c **(C)** HLA-DR **(D)**, CD36 **(E)** and CD56 **(F)** cell expression for each group (low (<1,000; n=51), medium (1,000-10,000; n=65) and high (>10,000; n=22) mean parasite density (per μl of blood)) was determined by flow cytometry and compared to endemic controls (EC). ^**^p ≤0.01 (ANOVA).

Previous studies have shown that *P. falciparum* infection is associated with changes in antigen presenting cell populations (APCs) [16]. Here, the percentage of CD14^+^ monocytes (precursor dendritic cells (DCs)/macrophage) in PBMCs for each group were examined. All groups displayed significantly lower percentages of CD14^+^ cells (<4%) compared to endemic controls (5-7%) (Figure 2A; *P* ≤0.01 for all infected groups). To dissect the APC subsets associated with malaria infection specific surface markers including HLA-DR (indicative of an active infection), CD11c (myeloid marker found on DCs), CD54 (also known as ICAM-1) (Intercellular Adhesion Molecule 1, is important for lymphocyte-APC binding and has been shown to be upregulated on APC during malaria infection) and CD36 (involved in phagocytosis of malaria infected red blood cells) were examined [17-21]. The percentage of CD54^+^ populations were associated with high density *P. falciparum* parasitaemia only (Figure 2B; *P* ≤0.01) while the percentage of CD11c^+^ populations were associated with medium (*P* ≤0.01) and high (*P* ≤0.01) but not low density *P. falciparum* parasitaemia (Figure 2C). No association was observed between HLA-DR^+^ (Figure 2D) and CD36^**+**^ cells (Figure 2E) and parasitaemia.

Since an increase in IFN-γ was not detected in plasma cells that are known to produce IFN-γ were examined to determine if there was a decrease in this population. Early IFN-γ is important in the control of *P. falciparum* parasitaemia and studies have shown that in the early stages of infection CD56^**+**^ natural killer cells (NK) and other leukocytes expressing the CD56 marker are good sources of IFN-γ [22]. The percentage of CD56^**+**^ cells in PBMCs were measured and these cells were positively associated with *P. falciparum* parasitaemia with significant increases in cell percentages for medium (*P* ≤0.05) and high density *P. falciparum* parasitaemia (*P* ≤0.05) (Figure 2F).

### Malaria infection was not associated with increased secretion of IFN-γ, TNF, IL-10 and IL-2 in mitogen-activated T-cell populations and no parasite specific immune responses were detected

**Figure 3:**
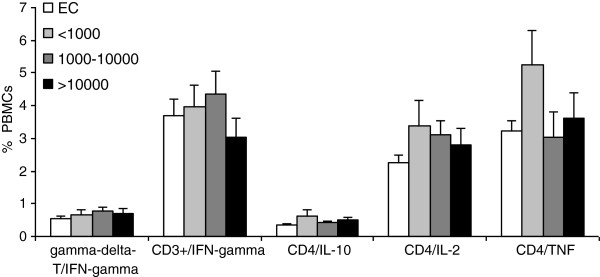
**Malaria infection was not correlated with an increase in the secretion of IFN-γ, TNF, IL-10 and IL-2 in mitogen-activated T-cell populations. **PBMCs were stimulated for 4 h with PMA and ionomycin. Cells were washed and stained for cell surface expression of γδTCR, CD3 and CD4 and were intracellular stained for IFN-γ, IL-2, IL-10 or TNF. Appropriate isotype controls were included to define parameter gates. PBMCs were gated on the lymphocyte population and the percentage of positive cells for each group (low (<1,000; n = 51), medium (1,000-10,000; n = 65) and high (>10,000; n = 22) mean parasite density (per μl of blood) were also compared to uninfected endemic controls (EC).

PBMCs were polyclonally activated with PMA and ionomycin and intracellular cytokine staining for IFN-γ, IL-10, IL-2 and TNF was performed. In order to evaluate the phenotype of the T cells present, cells were also stained for CD3, CD4, and γδ T-cells. All groups expressed significantly high levels of IFN-γ and TNF and low, but significant, levels of IL-10 and IL-2 following stimulation. There were no differences observed between the groups (Figure 3).

**Figure 4:**
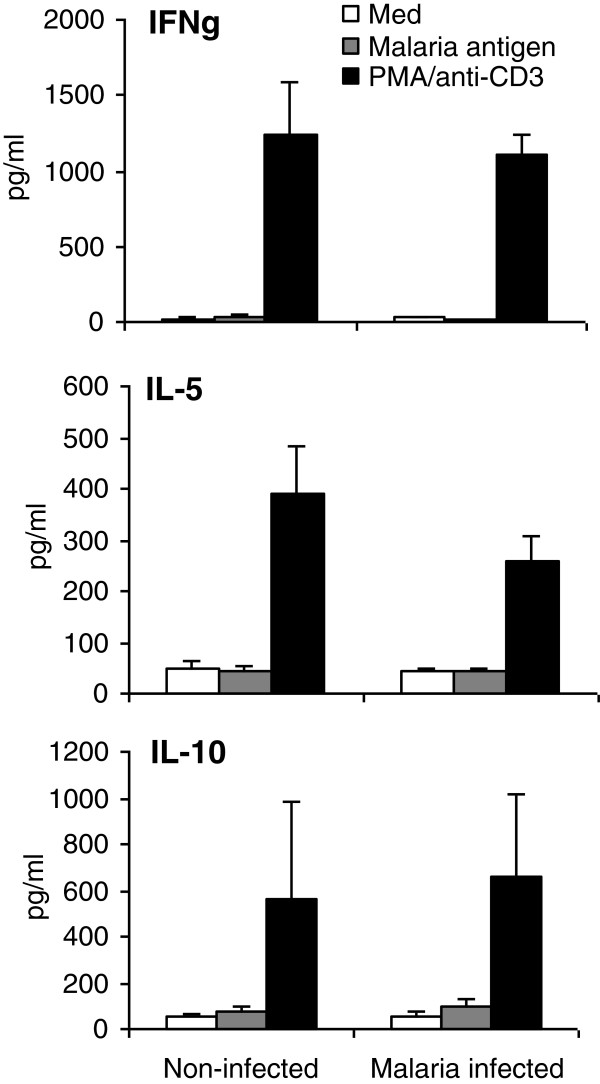
**PBMCs from infected individuals did not secrete antigen specific immune responses. **PBMCs (1x10^6^ cells/ml) from malaria infected (n=23) and non-infected (n=7) children were stimulated with medium (Med), mycoplasma free *P. falciparum* parasites (at a ratio of 1:5) (malaria antigen) and PMA/anti-CD3. After three days IFN-γ, IL-10, and IL-5 were measured in supernatant by commercial assay.

PBMCs (1×10^6^ cells/ml) from malaria infected (n=23) and non-infected (n=7) individuals were stimulated with mycoplasma free *P. falciparum* parasites (at a ratio of 1:5) and after three days IFN-γ, IL-10, and IL-5 were measured in supernatant. No parasite specific immune responses were detected while cPBMCs were capable of secreting all cytokines tested in response to our positive control PMA/anti-CD3 (Figure 4).

### Low intensity *Ascaris lumbricoides* infection did not impact upon *Plasmodium falciparum* parasitaemia or its associated immune responses

**Figure 5:**
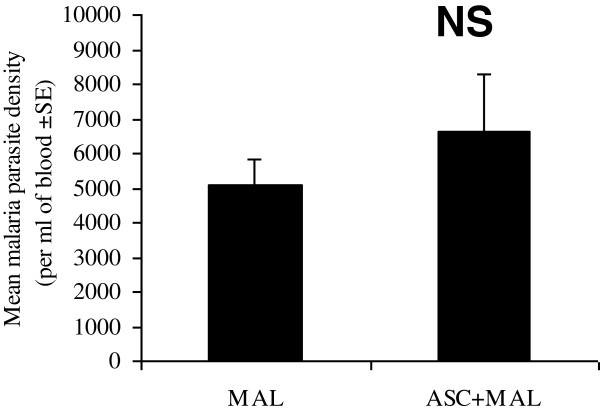
***Ascaris lumbricoides *infection did not impact upon *Plasmodium falciparum *parasitaemia. **Mean *P. falciparum* density (per μl of blood) was measured from malaria infected (MAL) and *Ascaris* and malaria co-infected (ASC+MAL) children by Giemsa stained blood slides (n = 109 for MAL; n = 32 for ASC+MAL) NS = non-significant.

**Figure 6:**
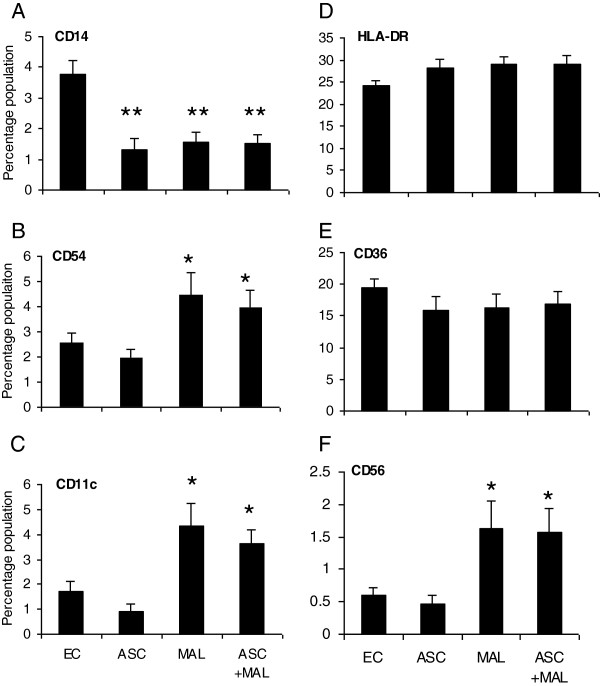
***Ascaris lumbricoides *infection did not alter the percentage of CD14**^**+ **^**CD11c**^**+**^**, CD54**^**+**^**, CD56 HLA-DR**^**+ **^**and CD36**^**+ **^**cell populations in-co-infected individuals. **PBMCs were stained extracellularly for CD14 **(A)**, CD54 **(B)** CD11c **(C)**, HLA-DR **(D)**, CD36 **(E)** and CD56 **(F)** cell expression for each group Ascaris infection only (ASC), Malaria infection only (MAL) and co-infected (ASC + MAL) was determined by flow cytometry and compared to endemic controls (EC). ^*^p ≤0.05; ^**^p ≤0.01 (ANOVA) and MAL group was compared to the ASC + MAL group (no significant differences).

**Figure 7:**
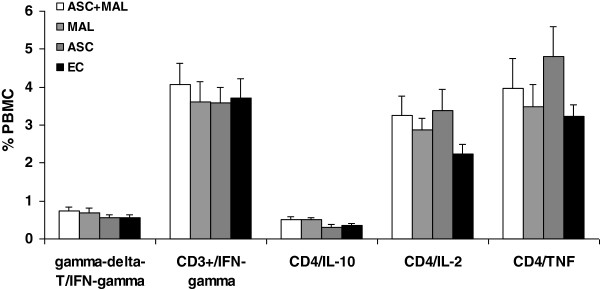
***Ascaris lumbricoides *infection did not alter IFN-γ, TNF, IL-10 and IL-2 expression in mitogen-activated T-cell populations. **PBMCs were stimulated for 4 h with PMA and ionomycin. Cells were washed and stained for cell surface expression of γδTCR, CD3 and CD4 and were intracellular stained for IFN-γ, IL-2, IL-10 or TNF-a. Appropriate isotype controls were included to define parameter gates. PBMCs were gated on the lymphocyte population and the percentage of positive cells for each group Ascaris infection only (ASC), Malaria infection only (MAL) and co-infected (ASC + MAL) was determined by flow cytometry and compared to uninfected endemic controls (EC) and MAL group was compared to the (ASC + MAL).

The data was reanalysed taking into account *Ascaris* infection (uninfected (n=69), *Ascaris* only (n=21), malaria only (n=109), *Ascaris* and malaria (n=32)). The prevalence of *A. lumbricoides* was 23% and all *A. lumbricoides*-infected children had a low intensity infection (<3,700 eggs per gram (epg)). *Plasmodium falciparum* percentages in the blood were calculated in the malaria-infected and co-infected individuals and no differences in *P. falciparum* parasitaemia were observed between the malaria-infected and co-infected groups (Figure 5). Furthermore, no differences were observed in immune responses between the malaria-infected and co-infected groups for all parameters tested (Additional file 3; Figures 6 and 7).

## Discussion

This study provides valuable insights into the immune responses associated with malaria infection in pre-school Nigerian children from 39–73 months. Studies looking at the immunological parameters in this age group are important given that the highest malaria mortality rates occur in children under five years [1]. Studies have shown that immune responses to malaria infection are established early in life and furthermore different ethnic groups respond differently to malaria infection and this is also established early in life [23]. There is little evidence of natural acquired immunity to malaria in children under two years as neonates less than 30 days old and children up to one year have reduced IFN-γ producing capacity [1]. No increase in IFN-γ levels was observed in the plasma of these children and this contradicts previous studies in children where increased levels of plasma IFN-γ were observed [1]. In addition, a number of other factors associated with active malaria infection were not observed in these children.

Significantly high levels of IL-10 in plasma was observed from malaria-infected children and this corresponded with a reciprocal decrease in IL-12p70 levels, similar to that reported in other studies [24]. Regression analysis revealed a positive association between *P. falciparum* parasitaemia and IL-10. IL-10 is a known antagonist of this pro-inflammatory cytokine [25] and recurrent malaria infection can induce an immunosuppressive environment through secretion of high levels of IL-10, thus inhibiting Th1 responses and facilitating parasite persistence [26]. Intracellular IL-10 was not detected in polyclonally stimulated T-cell subsets from malaria-infected children when compared to endemic controls. PBMCs from malaria infected children when stimulated with *P. falciparum* parasites did no exhibit antigen specific immune responses. Previous studies have reported that T-cell responses can be weak in young children infected with malaria and this lack of responses could explain why this age group is most susceptibility to infection [2].

The significant reduction in monocyte percentages in the infected groups compared to endemic controls was observed. Other reports have also shown a decrease in monocytes percentages during active parasitic infection [27]. During infection, circulating monocytes may be required at a higher rate to replenish resident macrophages and DCs. Alternatively, parasitic infections may induce monocyte apoptosis which could explain the observed decrease in monocyte percentages [28]. Despite the decreases in monocytes there was an increase in CD11c^+^ and CD54^+^ DCs in the infected individuals. The presence of predominantly mature CD11c^+^ DCs population may explain the lack of T-cell responses observed in young children as mature CD11c^+^ DC population lose its ability to phagocytose antigens which is necessary for presentation to effector and memory T-cells [17,18]. The increases in the percentage of CD54^**+**^ cells supports previous findings which demonstrated a link between increased CD54 expression and disease severity [28]. CD54 was previously shown to be upregulated on activated DCs, monocytes and other APCs during malaria infection in children and it is a known receptor that can bind *P. falciparum* erythrocyte membrane protein 1 [21]. CD11c and CD54 are expressed by many cell types and further analysis would shed light on the specific cell subset observed during infection.

Both γδ T cells and NK cell express CD56^+^ and this surface maker increased in the malaria-infected children. NK cells and γδT cells are thought to be critical in protection from malaria infection [29,30]. Depletion of these cells in murine malaria models has led to increased parasitaemia and delayed resolution of infection, emphasising their importance in early IFN-γ production [30,31]. While these cells are more likely to act as accessory IFN-γ secreting cells to effector T cells there was no increase in IFN-γ detected in the plasma of infected children. However, recently, these cells have also been shown to act as APCs and compensate for DCs in certain situations and further studies would be required to determine the role of this subset [31,32]. While an increase in CD56 cell population was observed, further studies are required to determine the CD56 population subset and examine if these cells secrete IFN-γ.

While the study region is endemic for *A. lumbricoides,* no differences in immune parameters were observed between the co-infected groups compared to the *P. falciparum*-infected group only. Perhaps no differences were observed because children had a low intensity *A. lumbricoides* infection, as was demonstrated in a previous report study by Nacher *et al.* (2000). Moreover, since infection resides in the gut, perhaps only the medium and high intensity infection can alter the immune response systemically. *Ascaris lumbricoides* infection can be protective in the more severe forms of *P. falciparum* infection [33] and since individuals with severe malaria infection were excluded from the study an association could not be determined.

## Conclusion

This data corroborates previous reports examining immune responses in malaria-infected children by showing that increases in IL-10 were positively associated with increased *P. falciparum* parasitaemia. Increases in innate immune cell populations that were previously associated with disease severity in malaria-infected individuals was also demonstrated [19,21,33]. Given that there are so few immunological studies in this age group, these findings could be useful in defining immune responses associated with increasing malaria parasitaemia in young children and therefore markers of disease susceptibility. It is estimated that 87% of children below the age of five are infected with malaria in the Osun State in south-western Nigeria [34], and validating these methodologies is important for future studies in this area. For example, birth to age five is an important range for the administration and study of many prophylactic paediatric vaccines and World Health Organization recommendations for routine immunization within this age group in Nigeria include both measles and yellow fever vaccines [35].

## Competing interests

GlaxoSmithKline sponsored the drug albendazole, which was used in larger clinical trial. The authors declare that they have no competing interests. The authors also declare that they have no financial competing interests.

## Authors’ contributions

CN carried out all immunological experiments and drafted the manuscript. MP is our biostatistician and performed the statistical analysis. DJD provided assistance in the immunological assays, AA provided assistance in the immunological assays, PK collected clinical samples and provided the parasitological data, SFM collected clinical samples, SOA participated in the design of the study, CH conceived the study, and participated in its design and coordination. SMO’N conceived the immunological aspects of study, participated in its design and edited the manuscript. All authors read and approved the final manuscript.

## Supplementary Material

Additional file 1 **Sample of gating strategy for T Cells. **Shown is a PBMC sample stimulated with 50 ng/ml PMA and 1 mg/ml ionomycin for 4 h in the presence of BFA (10 mg/ml). Cell subsets were identified as CD3 cells and then analysed for expression of cytokines and markers γδcells. Click here for file

Additional file 2 The average age, parasitemia, cytokine, nitric oxide (NO), RANTES, metalloproteinase (MMP) type 8 and tissue inhibitor of metalloproteinase (TIMP) type 1 plasma concentrations from the study cohort classified according in infection status (uninfected, *Ascaris *only, malaria only, *Ascaris *and malaria. Click here for file

Additional file 3 The average age, cytokine, nitric oxide (NO), RANTES, metalloproteinase (MMP) type 8 and tissue inhibitor of metalloproteinase (TIMP) type 1 plasma concentrations from the study cohort classified according to *P falciparum *parasitaemia. Click here for file
