# Supplementary figures and images for: Plasma cytokines, chemokines and cellular immune responses in pre-school Nigerian children infected with Plasmodium falciparum
Source: Malar J. 2013 Jan 7;12:5. doi: 10.1186/1475-2875-12-5 (PMC3545738; doi:10.1186/1475-2875-12-5)

## Slide 1
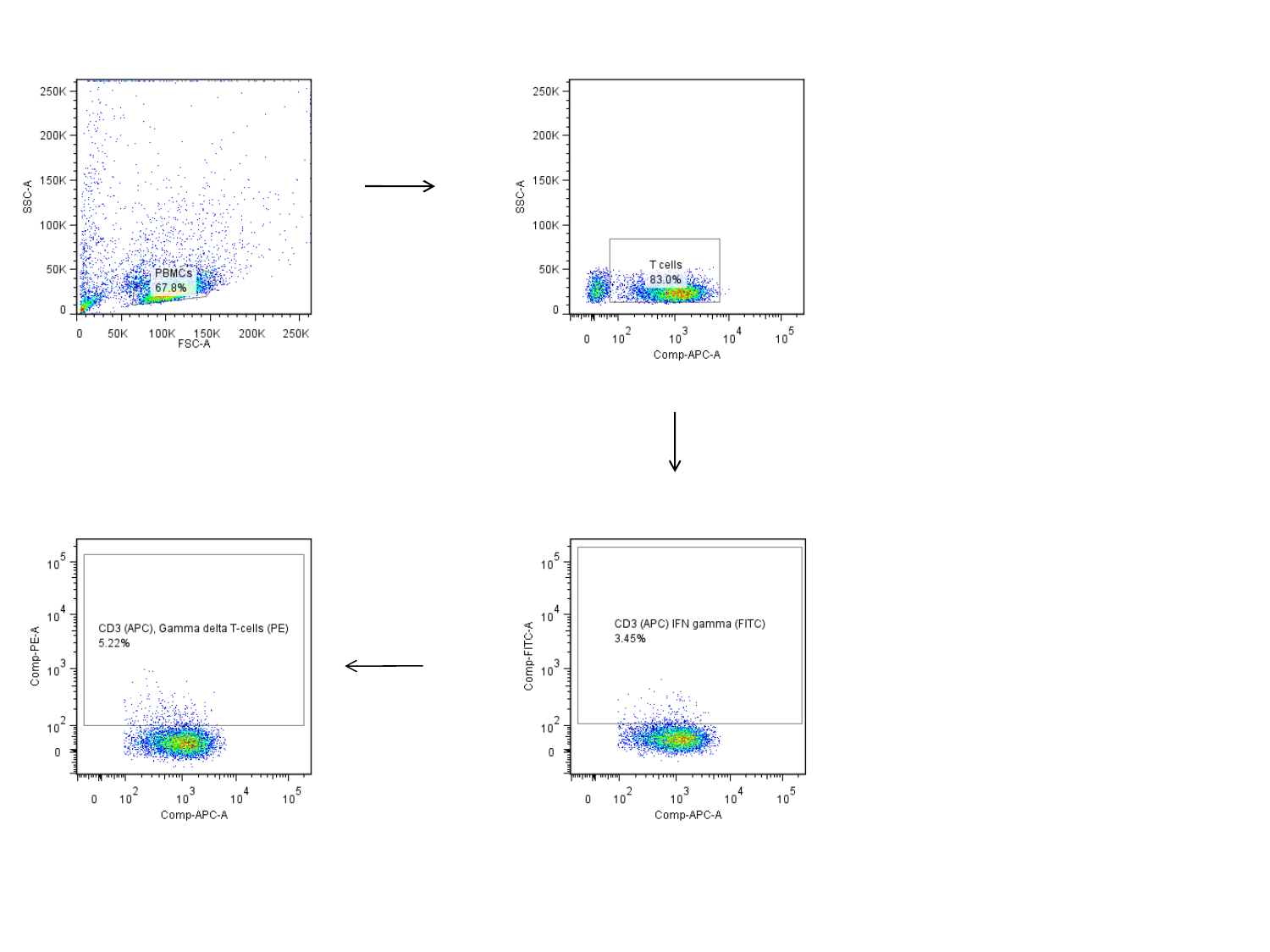

Supplement: Additional file 1 — Sample of gating strategy for T Cells. Shown is a PBMC sample stimulated with 50 ng/ml PMA and 1 mg/ml ionomycin for 4 h in the presence of BFA (10 mg/ml). Cell subsets were identified as CD3 cells and then analysed for expression of cytokines and markers γδcells. [file 1475-2875-12-5-S1.ppt]
