# Supplementary material for: Plasma cytokines, chemokines and cellular immune responses in pre-school Nigerian children infected with Plasmodium falciparum
Source: Malar J. 2013 Jan 7;12:5. doi: 10.1186/1475-2875-12-5 (PMC3545738; doi:10.1186/1475-2875-12-5)
Supplement: Additional file 2 — The average age, parasitemia, cytokine, nitric oxide (NO), RANTES, metalloproteinase (MMP) type 8 and tissue inhibitor of metalloproteinase (TIMP) type 1 plasma concentrations from the study cohort classified according in infection status (uninfected, Ascaris only, malaria only, Ascaris and malaria. [file 1475-2875-12-5-S2.doc]

**Table 1.** The average age, parasitemia, cytokine, nitric oxide (NO), RANTES, metalloproteinase (MMP) type 8 and tissue inhibitor of metalloproteinase (TIMP) type 1 plasma concentrations from the study cohort classified according in infection status (uninfected, *Ascaris* only, malaria only, *Ascaris* and malaria.

|  | **Uninfected endemic** | **Ascaris** | **Malaria** | **Ascaris/Malaria** |
| --- | --- | --- | --- | --- |
| **Children** | 69 | 21 | 109 | 32 |
| **Mean age (months)** | 46 | 37 | 45 | 45 |
| **Mean *A lumbricoide*s (epg)** | 0 | 2577 ± 745.0 | 0 | 2889.0 ± 807.7 |
| **Mean Parasite density (per μl of blood)** | 0 | 0 | 5097 ± 717.4 | 6618.7 ± 1683.2 |
| **IFNγ (pg/ml)** | 67.7 ± 23.5 | 33.1 ± 8.4 | 71.2 ± 27.9 | 32.3 ± 5.0 |
| **TNF (pg/ml)** | 181.3 ± 11.2 | 144.0 ± 13.3 | 193.2 ± 8.7 | 188.9 ± 16.2 |
| **IL-4 (pg/ml)** | 117.5 ± 8.8 | 85.6 ± 15.9 | 123.1 ± 6.1 | 115.1 ± 12.1 |
| **TGFβ (pg/ml)** | 8688.2 ± 898.1 | 7046.3 ± 1435.6 | 8469.5 ± 637.5 | 6471.9 ± 1153.2 |
| **IL-17 (pg/ml)** | 5.1 ± 3.5 | 1.0 ± 0.6 | 1.6 ± 0.4 | 1.8 ± 1.1 |
| **NO (mM)** | 6.6 ± 0.8 | 7.4 ± 0.9 | 6.5 ± 0.5 | 5.6 ± 0.5 |
| **RANTES (pg/ml)** | 617.2 ± 20.9 | 639.6 ± 37.3 | 576.1 ± 15.8 | 613.2 ± 33.3 |
| **MMP8 (pg/ml)** | 1468.6 ± 116.4 | 1379.0 ± 184.4 | 1606.3 ± 84.7 | 1569.1 ± 163.9 |
| **TIMP1 (pg/ml)** | 2406.1 ± 70.8 | 2539.5 ± 81.0 | 2434.4 ± 54.6 | 2577.0 ± 103.1 |
| **IL-12p70** | 473.3 ± 33.4 | 417.8 ± 63.4 | 363.8 ± 22 | 361.9 ± 46.1 |
| **IL-10** | 155.5 ± 9.3 | 99.2 ± 14.5 | 203.8 ± 8 | 203.7 ± 29.5 |

Results are mean ± SEM.
