# Supplementary material for: Plasma cytokines, chemokines and cellular immune responses in pre-school Nigerian children infected with Plasmodium falciparum
Source: Malar J. 2013 Jan 7;12:5. doi: 10.1186/1475-2875-12-5 (PMC3545738; doi:10.1186/1475-2875-12-5)
Supplement: Additional file 3 — The average age, cytokine, nitric oxide (NO), RANTES, metalloproteinase (MMP) type 8 and tissue inhibitor of metalloproteinase (TIMP) type 1 plasma concentrations from the study cohort classified according to P falciparum parasitaemia. [file 1475-2875-12-5-S3.doc]

**Table 2.** The average age, cytokine, nitric oxide (NO), RANTES, metalloproteinase (MMP) type 8 and tissue inhibitor of metalloproteinase (TIMP) type 1 plasma concentrations from the study cohort classified according to *P falciparum* parasitaemia.

| **Mean Parasite density (per μl of blood)** | **Uninfected** | **<1000** | **1000-10000** | **>10000** |
| --- | --- | --- | --- | --- |
| **Children** | 89 | 51 | 65 | 22 |
| **Mean age (months)** | 35 | 40 | 44 | 36 |
| **IFNγ (pg/ml)** | 60.2 ± 18.2 | 42.0 ± 6.8 | 94.4 ± 52.2 | 42.2 ± 8.9 |
| **TNF (pg/ml)** | 173.1 ± 9.3 | 191.2 ± 11.3 | 202.2 ± 11.7 | 187.95 ± 22.2 |
| **IL-4 (pg/ml)** | 109.4 ± 7.7 | 127.85 ± 8.4 | 113.95 ± 7.3 | 118.5 ± 17.1 |
| **TGFβ (pg/ml)** | 8237.1 ± 761.9 | 8832.2 ± 940.89 | 7786.4 ± 785.8 | 6877.1 ± 1478.2 |
| **IL-17 (pg/ml)** | 4.1 ± 2.7 | 0.8 ± 0.24 | 2.3 ± 0.60 | 2.7 ± 1.5 |
| **NO (mM)** | 6.9 ± 0.7 | 6.6 ± 0.6 | 5.7 ± 0.6 | 7.0 ± 1.4 |
| **RANTES (pg/ml)** | 623.7 ± 18.2 | 565.7 ± 21.5 | 598.6 ± 19.5 | 613.9 ± 45.95 |
| **MMP8 (pg/ml)** | 1465.6 ± 97.97 | 1488.9 ± 114.3 | 1686.7 ± 102.2 | 1890.5 ± 209.1 |
| **TIMP1 (pg/ml)** | 2432.9 ± 57.7 | 2470.7 ± 80.4 | 2427.9 ± 74.2 | 2543.2 ± 110.2 |
| **IL-12p70** | 483.7 ± 29.5 | 397. ±9 25.7 | 340.9 ± 25.7 | 336.2 ± 40.8 |
| **IL-10** | 142.4 ± 8.3 | 178.3 ± 9.5 | 207.1 ± 11 | 283.8 ± 37.1 |

Results are mean ± SEM.
